# “The research assistants kept coming to follow me up; I counted myself as a lucky person”: Social support arising from a longitudinal HIV cohort study in Uganda

**DOI:** 10.1371/journal.pone.0262989

**Published:** 2022-01-25

**Authors:** Jeffrey I. Campbell, Angella Musiimenta, Sylvia Natukunda, Nir Eyal, Jessica E. Haberer

**Affiliations:** 1 Boston Children’s Hospital, Boston, Massachusetts, United States of America; 2 Department of Information Technology, Mbarara University of Science and Technology, Mbarara, Uganda; 3 Mbarara University of Science and Technology, Mbarara, Uganda; 4 School of Public Health, Rutgers University, Piscataway, New Jersey, United States of America; 5 Department of Philosophy, Rutgers University, New Brunswick, New Jersey, United States of America; 6 Center for Population-Level Bioethics, Rutgers University, New Brunswick, New Jersey, United States of America; 7 Massachusetts General Hospital, Boston, Massachusetts, United States of America; Children’s Mercy Hospitals and Clinics Department of Pathology and Laboratory Medicine, UNITED STATES

## Abstract

**Background:**

Participation in longitudinal research studies in resource-limited settings often involves frequent interactions with study staff and other participants, as well as receipt of incentives and transportation reimbursements. Social support—receipt of material and emotional resources from one’s social network—has been linked to antiretroviral adherence in sub-Saharan Africa. The extent to which social support arises from study participation, its range and depth, and its implications for observational study conduct, have not been extensively described.

**Methods:**

We conducted individual open-ended and semi-structured interviews with participants in a longitudinal, observational antiretroviral therapy adherence monitoring study in Mbarara, Uganda. Participants were asked about their experiences in the longitudinal study and their interactions with research staff. We also interviewed study research assistants (RAs). Deductive and inductive coding were used to identify content related to the experience of study participation. Codes were organized into themes, and relationships between themes were used to develop overarching categories.

**Results:**

Sixty longitudinal study participants and 6 RAs were interviewed. Instrumental and emotional social support emerged as pervasive and valued aspects of longitudinal study participation. Instrumental support that participants received consisted of enhanced linkage to medical care, health education, and direct and indirect material benefits. Emotional support consisted of perceptions of feeling “cared for” and social interactions that permitted escape from HIV-related stigma. Both instrumental and emotional support often arose through the close relationships participants formed with research staff and with each other. Study-derived social support motivated some participants to adhere to antiretroviral therapy—an unanticipated effect potentially influencing the longitudinal study’s primary observational outcome.

**Conclusions:**

Longitudinal study participation resulted in instrumental and emotional social support for participants. The depth of support participants experienced has implications for observational study design in resource-limited settings, including need to assess potential effects on study outcomes; consideration of social support during risk/benefit assessment in study ethics review and consent; and vigilance for consequences of social support loss when studies end.

## Introduction

For people living with HIV (PLWH) in resource-limited settings, participation in longitudinal health studies often involves a series of interpersonal interactions and exchange of material resources and information. Repeat interactions with research staff occur in clinical settings, through mobile phones, in participants’ homes, and in the community. Others have documented close relationships that can form between study participants and research staff in sub-Saharan Africa, and the ethical and practical challenges that these relationships can create [1–5]. Participants may encounter one another during study visits or at study-wide events [6]. Participants also usually receive reimbursement for study-related activities (e.g. transportation) and compensation (e.g. incentives allocated during study visits) [7]. The manner in which participants ascribe value to their relationships with study staff and other participants, and perceive of reimbursements and compensation, has been incompletely examined. Yet it is essential to understand how these relationships and material exchanges shape enrollment, retention, study-related behaviors, data collection, and data interpretation. Moreover, for PLWH who participate in research, understanding how the research environment creates and facilitates social interaction and exchange can provide insight into these individuals’ health and wellbeing.

Social support refers to the provision of psychological and material resources by people within one’s social network [8]. Research from resource-limited settings has explored how individuals commonly depend on others for support and engage in community-level resource sharing [9]. Studies from these settings have shown that social support can arise from a variety of sources, such as from friends and family, as well as from communities of people who share common conditions like HIV [10].

In Uganda, where our team was involved in a longitudinal antiretroviral therapy (ART) adherence monitoring study from 2005–2015, social support has been shown to help people living with HIV (PLWH) overcome barriers to receiving HIV-related care, such as chronic food insecurity and the cost of transport to clinics [11–13]. Receipt of social support has also been linked to decreased HIV-related stigma [8] and improved ART adherence [14]. Meanwhile, increased availability of mobile phones has made access to health-related social support more available to individuals receiving long-term therapies in Uganda [15].

In this paper, we examine how participants in a longitudinal ART adherence monitoring study derived social support from study participation, the ways social support was tied to study-related relationships, and the implications study-related social support has for research conduct and data interpretation.

## Methods

We conducted a qualitative study of participants enrolled in a longitudinal cohort study that monitored adherence to ART in southwest Uganda, and of research assistants (RAs) for the study. Our primary aim was to understand ethical considerations related to adherence monitoring, which we have described elsewhere [16–18]. In this manuscript, we analyze social support that participants derived from study participation, which was an emergent and unexpected finding of our study.

### Parent study

The Uganda AIDS Rural Treatment Outcomes (UARTO) study (NCT01596322) was a longitudinal, observational cohort study of ART adherence in Mbarara, Uganda—a city in the southwestern part of the country. All participants received HIV-related care and free ART through the Mbarara Regional Referral Hospital Immune Suppression Syndrome (ISS) Clinic [19]. In the UARTO study, electronic adherence monitors (EAM) were used to characterize ART adherence. UARTO participants were permitted to participate in UARTO even if they opted to not use the EAM following enrollment, meaning that there was a subset of UARTO participants who were EAM non-users.

UARTO participants were typically seen in the ISS clinic every one to three months, per recommendation from their treating clinicians. They also attended UARTO study visits in a building adjacent to the clinic every three to four months. During study visits, they completed questionnaires and provided biological specimens. They were given a meal while awaiting study procedures and received an incentive at each study visit—usually a bar of soap or a kilogram of sugar. Transportation reimbursement was also provided based on self-reported cost of travel to and from the clinic. The UARTO study team organized dissemination events during the study, at which participants gathered to hear about results and receive thanks for their participation [6].

UARTO RAs, who were university-educated residents of Mbarara, served as the main points of contact for study participants. All RAs were trained in ethical research practice and data collection. RAs generally followed a set of participants for months to years, meaning that participants repeatedly interacted with the same RA during the study. RAs performed study procedures (e.g., coordinating tests, administering questionnaires) when participants came to scheduled study visits. Additionally, RAs made field visits to participants’ homes or other pre-specified locations (e.g. when participants were concerned that home visits could result in status disclosure) to draw blood in instances of non-adherence and to assess device functionality. Because UARTO was designed to be observational, RAs were advised to refer health-related questions to the clinic staff.

### Study design and participants

We conducted individual qualitative interviews with UARTO study participants and RAs. Because our overarching goal was to explore ethical considerations surrounding EAM use, we used a criterion-based purposive stratified sampling approach to recruit UARTO participants who did and who did not use EAMs. All participants were ≥18 years old, currently receiving ART, and had been enrolled in UARTO for at least 6 months. We recruited 40 participants who were EAM users, and 20 participants who were EAM non-users; these pre-specified sample sizes were selected because they were anticipated to be sufficient to achieve thematic saturation [20, 21]. UARTO RAs identified key informants who they thought would be able to provide diverse and rich insights about EAM use. Interview participants were recruited by phone or in person during UARTO study visits. The study was explained to participants as a way to understand their experiences with EAMs. All individuals who were approached to participate in interviews agreed to enroll in our study.

Interviews with UARTO participants were completed in two phases. First, we conducted exploratory interviews to elicit general impressions surrounding EAM use and UARTO study participation (n = 20 EAM users). Second, we conducted focused interviews to investigate concepts derived from ethics theory [22] and from exploratory interviews (n = 20 EAM users and 20 EAM non-users). We only recruited EAM users in the exploratory phase of our study, due to relatively low number of EAM non-users in the UARTO study. EAM users who participated in exploratory interviews were not included in focused interviews. In addition, we conducted in-depth interviews with UARTO RAs (n = 6), to contextualize participants’ interviews. Interview guides are provided in **S1 File**.

Thematic saturation was assessed during review of interviews as the interviewing process was ongoing, and again during analysis of themes following completion of interviews. Based on these assessments, we determined that we had achieved thematic saturation.

### Data collection

We interviewed UARTO participants and RAs between August 2014 and June 2015. After consenting to participate, all participants completed brief, standardized demographic questionnaires, which were entered into a secured electronic database using Research Electronic Data Capture (REDCap) version 6 [23].

A Ugandan researcher trained in qualitative interviewing, who was not known to participants before the study, conducted all interviews in Runyankole, the predominant language in Mbarara. All interviews took place in a private location near the main UARTO research site and were digitally recorded. Interviews were then simultaneously translated and transcribed into English by the same Ugandan researcher, and were reviewed and discussed with an American researcher at time of transcription to ensure a shared concept of key terms and ideas.

An American researcher trained in qualitative interviewing, who was known to RAs before the interviews but who was not directly involved in the UARTO study, conducted interviews with RAs in English in a private research office. Interviews were transcribed in English.

### Data analysis

We performed an initial inductive content analysis of exploratory interviews [24]. Participants in exploratory interviews described social support gleaned from involvement in UARTO. Because the finding of perceived social support was unanticipated, we further explored this concept in depth in focused interviews. We used both inductive and deductive content analysis approaches to explore focused interviews.

Initial analysis of both exploratory and focused interviews began with review and discussion of 20% of transcripts by American and Ugandan researchers. Our initial transcript review aimed to identify content relevant to the primary ethical questions the study sought to answer, and captured emergent themes related to the experience of study participation. Content was then organized as codes in a codebook; initial codes captured data about the primary ethical questions explored during interviews, as well as about emergent themes that arose in initial transcript review. Codebook development was iterative and involved defining codes and identifying representative quotes from transcripts to illustrate these codes. Codes were reviewed for common content and themes, and were nested or merged when they were deemed to represent overlapping concepts. The final codebook was then imported into NVivo version 11, and two American researchers coded all interviews. Discrepancies in coding were resolved by discussion among the team. The process of developing codes, the codebook, and content identification was performed separately for exploratory and for focused interviews.

Following coding of all transcripts, codes were organized into categories in an iterative process that involved review of all coded text. Themes were derived by identifying relationships between categories. Organization of themes into social support categorizations and classifications was conducted during a series of meetings involving all members of the team. During these discussions, salient themes were identified as those that exemplified specific experiences of social support, including emotional support and material exchange. We compared responses of EAM users from EAM non-users within codes and categories, and found that EAM users and non-users experienced social support arising from study participation similarly. Results from these two groups are therefore presented together throughout the text.

### Ethical review

The institutional review board at Partners Healthcare (through Massachusetts General Hospital), the research ethics committee at the Mbarara University of Science and Technology, and the Uganda National Council of Science and Technology reviewed and approved this study. All participants provided written informed consent prior to participation. The study and consent form were explained to participants in detail, and the consent form was read to participants with limited literacy. The consent process was conducted in Runyankole with UARTO participants, and in English with RAs.

## Results

We interviewed 60 UARTO study participants, of whom 41 (68%) were women. Median age was 42 years (interquartile range: 38–47) (**Table 1**). Most participants were literate (53 [88%]), and 20 (33%) had completed education beyond primary school. We additionally interviewed 6 UARTO RAs. Median age of RAs was 36 (interquartile range: 34–38), and 4 were female.

**Table 1:** Participant characteristics.

UARTO Participants	EAM Users (n = 40)	EAM Non-Users (n = 20)
Age, median (IQR)	41 [35–46]	45 (42–50)
Female, n (%)	28 (70%)	13 (65%)
Literate, n (%)	36 (90%)	17 (85%)
Education Level, n (%)		
Never attended school	4 (10)	2 (10)
Primary	23 (57.5)	11 (55)
O-Level*	9 (22.5)	3 (15)
A-Level*	1 (2.5)	3 (15)
University/vocational	3 (7.5)	1 (5)
Post-graduate	0 (0)	0 (0)
Socioeconomic Status		
Earns a salary, n (%)	8 (20%)	5 (25%)
Monthly salary, median (IQR) (USD)†	$65 ($44-$130)	$116 ($87-$116)
Monthly non-salaried income, median (IQR) (USD)†	$12 ($4-$36)	$63 ($24-$81)
Monthly household expenditures, median (IQR) (USD)†	$75 ($39-$148)	$87 ($22-$171)
Time from UARTO enrollment to interview, mean (SD) (years)	5.5 (2.7)	8.0 (1.1)
**UARTO Research Assistants (n = 6)**		
Age, median (IQR)	36 [34–38]	
Female, n (%)	4 (66)	

Abbreviations: UARTO–Uganda AIDS Rural Treatment Outcomes study; EAM–electronic adherence monitor; IQR–interquartile range; USD–United States Dollar; SD–standard deviation; CAB–Community Advisory Board; REC–Research Ethics Committee.

* In the Ugandan education system, O-Level indicates completion of four-year secondary school (often attended by 13–16 year olds). A-Level is two-year pre-university schooling (often attended by 17–18 year olds).

† Monetary conversion calculated at 1 US Dollar = 3445 Ugandan Shillings (as of January 12, 2015).

### Overview of qualitative findings

**Fig 1:**
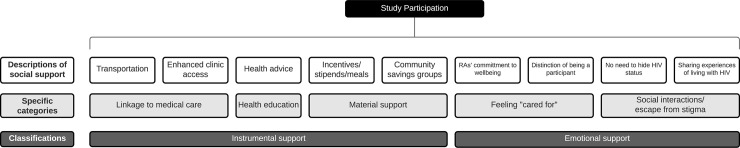
Categorization of social support arising from study participation.

Participants described receiving multiple types of social support as a result of participating in the UARTO study. As shown in **Fig 1**, we broadly classified experiences of linkage to medical care, receipt of material support, and health education as instrumental support. We classified perceptions of feeling “cared for”, social interactions, and escape from stigma that participants experienced as emotional support. Participants highly valued the social support received through the study, which often motivated adherence behaviors. Descriptions of social support did not differ between EAM users and non-users.

### Instrumental support

UARTO study staff connected participants to medical care unrelated to study participation. For example, although not a part of the study’s design, participants leveraged relationships with RAs to get transportation to the hospital and to access clinical care:

*For example*, *there is a time I became sick at home and I called [RA’s name] on the phone and told him that I am sick*. *He drove and came*. *He took me to the clinic for the staff*, *they took off blood from me and he took me back home*. *He told me that he would bring me drugs [ART] and the results after*. *If I had not enrolled in the study I would not have met someone like [RA]*. (Female EAM-user, age 39)

RAs also aided participants to access the ISS clinic and the hospital more efficiently. For instance, RAs facilitated transportation to and from the hospital, welcomed participants, and enabled participants to bypass lines and avoid long wait times at the clinic:

*I like being involved in it [UARTO] because we never used to [wait] in the line even to get our CD4*. *It helps me a lot when I come for my medication*: *even if am not feeling well my RA helps me get medication*, *by getting for me my file*, *he weighs me*, *he presents it there and helps me get my drugs*. *He even helps me to get a motorcycle or a vehicle back home or at time he even drops me at home when am not feeling well*. (Female EAM-user, age 51)

During routine research visits, participants and RAs frequently talked about health and healthcare unrelated to the UARTO study. Participants often viewed RAs as health educators and advisors:

*When they come*, *they visit you and counsel you*, *they teach you and give you advice*. *They advise us to sleep under a mosquito net*, *if you are married you should all be tested and things like that*. *They ask if we want to have children*, *they tell us the foods we should eat to be healthy and to take water*. *So*, *I like all these pieces of advice*. (Female EAM-user, age 30)

*They [RAs] listen to our problems and they tell us how to avoid that*. *They normally advise us*, *like for me I used to be a smoker*, *but my RA helped me to quit smoking*, *so they are good to us and they help us to quit those bad habits*. (Male EAM non-user, age 50)

An RA provided another example:

*Yes at times you realize that they want to confide in us their secrets*. *For example someone tells you that she is pregnant but she does not want the husband to know*. *So they seek your advice*. (RA 1)

Participants also explained that participation in UARTO helped them understand the value of ART adherence. This educational messaging, which was not a part of the UARTO protocol, became an ancillary feature of study participation and “encouraged” some participants to adhere to ART:

*She [RA] keeps telling me to think about how I used to be and how am now*, *and this encourages me to keep taking my drugs well so that my health can be good*. *She emphasizes to me the importance of taking my drugs and tells me to see it as something very important*. (Female EAM non-user, age 42)

Corroborrating these observations, one RA noted:

*I think they have gained knowledge and self confidence and responsibility from the questions we ask them in the study*. (RA 2)

Participants viewed transportation stipends, incentives (soap, sugar), and meals that UARTO provided as material support, which was at risk when the study closed:

Respondent (R): *I like everything about it in fact when they told us that its ending I felt very bad because they have been offering me counselling and support*.

Interviewer (I): *Tell me more about this support*?

R: *They have been giving me transport refund whenever I have been coming here to change the battery [of the EAM] or to do a blood draw they have been also giving me a kilo of sugar a bar of washing soap*. (Female EAM-user, age 39)

This material support contributed to the relationships between research staff and participants. Participants explained that the incentives distributed by RAs represented the UARTO study’s tangible commitment to their wellness:

R: *I like it because I have not seen any problem with it especially for us as women*. *At times we can be there hard up and they come and bring us some support*.

I: *Like what support*?

R: *Like soap and sugar*

I: *Anything else*?

R: *Even RAs coming to visit us give us the courage to feel that someone cares about you*. (Female EAM-user, age 31)

*They give us soap*, *transport; they give us breakfast because we come early*. *It’s good breakfast*…*Breakfast is something that encourages us to come because if you come before taking breakfast you are sure you will get one*, *if you have borrowed transport to come you are sure they will refund it and you will not go back worried about refunding it*. (Male EAM-user, age 49)

Additionally, participants expressed that the incentives were a reflection of being treated well by the research staff:

*[RA’s name] is a good person*. *She used to come and bring me incentives and she would send me a car to pick me at times*, *she would even also interview me at times*. *She brings me anything that is there for us in the project like sugar and soap*. *She gave me a phone*, *the device [EAM] and incentives so I have not missed anything from the study and yet there are other people who are greater than me and who wanted to be in the study and get these things I mentioned but they did not get the chance*. *So I thank my RA she is a good person and our relationship is good*. (Female EAM-user, age 45)

UARTO participantation fostered communities of PLWH that enabled participants to provide instrumental support to each other. For instance, one participant described a savings group that included other UARTO participants. This savings group functioned as a safety net, providing transportation funds to meet extra costs of care:

I: *Tell me about your involvement in the UARTO Study*.

R: *It helps me to associate with other people who are HIV positive and this helps me to learn that am not alone and I get encouraged*. *For example in my area we are 157 people living with HIV*. *Some of them are in UARTO study while others are not but they are getting their medication from Mbarara Hospital*. *We formed our savings group where each one of us saves 5000 Uganda shillings only [approximately $1*.*70 US] per month and this money is used to help in case a member needs to come to Mbarara for medical care and they do not have transport*. *So being involved in the study has helped me to associate with others*…*we know each one’s return date and we follow each other up to go for review and in case if one has no transport we provide it from the pool of money that we contribute per month*. (Male EAM-user, age 58)

In sum, multiple forms of instrumental support were highly valued by study participants and acted as meaningful motivation for self-care, including adherence to ART (i.e., the observational goal of the study).

### Emotional support

Participants felt that RAs’ diligence in conducting study procedures demonstrated “care” and a commitment to participants’ long-term well-being:

*…I know if I do not swallow they will know and call me*… *I know that shows that they care about me because otherwise if they do not care*, *they would just leave me*. (Female EAM-user, age 32)

*I used to feel bad that I have HIV but when I joined the study they used to come and pick me and drop me*. *This showed that they care about me*. (Female EAM-user, age 45)

The sentiment of caring was closely linked to close relationships and friendship with research staff:

I: *Tell me the ways in which this device [EAM] helps you*.

R: *It keeps well my drugs and it keeps my secret*.

I: *Anything else*?

R: *It unites me with the health workers who are taking care of me*. *It has also increased my friends like you; you have been added on my list of friends*. *It is not a joke that someone cares to call you and you talk about your life*. (Female EAM-user, age 30)

An RA explained:

*Actually they [participants] look at us as more than RAs*. *They look at us as someone to solve all their problems*. *Actually [one participant] called me and said [RA’s name] you have taken long without visiting me*. *You should find some time and come and visit me…*.*Knowing that you are there also somehow helps them to take their medication*… (RA 3)

Likewise, participants felt that RAs were concerned about their health, which motivated them to adhere to ART:

*[RA] used to come and visit me and check on the device [EAM]*. *She would ask me how am I feeling so I would see that she feels concerned about me*. *Now if someone is concerned about you like that*, *why you shouldn’t also try to be concerned to take your drugs well*. *So I see their [RAs’] responsibility is that they visit us and encourage us to take our drugs well and in time*. *Our interaction with the researchers is really good*. (Female EAM non-user, age 52)

Participation in the study was also perceived as a positive distinction among other clinic attendees:

*…They [the study] picked me out of many people and I feel special*. *In fact others complain why they care about us who are in the study more*. *So this gives me a feeling of connection to the clinic*. (Male EAM-user, age 38)

Through participation in UARTO, participants frequently interacted with study staff and other research participants (e.g., at study clinic visits) who were aware of their HIV serostatus. This community of staff and study participants attenuated HIV-related stigma:

*…when I joined the study it helped me take away stigma because it helped me to disclose to some people I never would have disclosed to but when I met them in the study it helped me to disclose to them*. *When I come to the clinic I also see people who are looking good and so I know that if I continue taking my drugs I will be like them*. (Male EAM non-user, age 47)

An RA noted:

*…they have enjoyed interaction with different people in the study and people in the clinic and it has reduced sigma*. (RA 2)

Study visits provided an opportunity for participants to share information and interact informally. Participants gained emotional support and encouragement by sharing experiences of living with HIV in their communities. Encouragement often took the form of health advice, given participants’ preoccupation with health and wellbeing:

*If they tell me that it [CD4 count] has reduced I feel very worried but when I share my worry with other participants on the bench [at the clinic]; they encourage me and advise me on how I can increase my CD4 count*…*basing on their experience*, *what foods to eat like fruits and vegetables*. (Female EAM-user, age 22)

*It has been good to me because it put in me confidence*, *it put in me self-esteem and I found there other colleagues who gave me their testimonies of how they have improved because I was not among the first people to join but I got confidence that I must live and we are living*. (Male EAM-user, age 49)

Together with the instrumental support achieved through study participation, emotional support from RAs and other participants bolstered self-worth, and motivated ART adherence and achievement of good health.

## Discussion

In this qualitative study of participants enrolled a long-term observational ART monitoring study and the RAs working closely with them, we learned of the importance of social support that participants gained through longitudinal study participation. Support was both protocol-directed (e.g., transportation stipends and study incentives) and unintended by the study (e.g., transport to the clinic, health advice, and access to a savings group). Unintended support derived primarily from close relationships that formed during repeated interactions with research staff and frequent connections with other study participants. Although broadly characterized social support has previously been identified as arising from study participation in sub-Saharan Africa [25], our results enable a contoured understanding of the origins, categories, and depth of support. These insights have implications for 1) investigators seeking to understand sources of bias in their data and the social context from which their data arise, 2) ethics boards aiming to assess recruitment, consent, and cost/benefit ratios of studies, and 3) participants who are fundamentally affected by support they glean through engagement in research.

Participants perceived linkage to healthcare as a major source of study-derived support. Enhanced access to clinical services arose both through transportation to the clinic and through streamlined navigation of the clinical intake and medication dispensing process. Other qualitative studies of research participants in sub-Saharan Africa have identified healthcare access as a driver of study enrolment and retention. In a mixed-methods sub-study of a large trial in South Africa and Kenya that sought to explain trial participants’ low adherence to HIV pre-exposure prophylaxis (PrEP), 93% of participants indicated that they continued to participate in the trial because of the ancillary healthcare benefits the trial provided, despite not wanting to use PrEP [26]. Kingori details the “empty choice” that research participants in sub-Saharan Africa may face when given the option to participate in research, due to improved medical care and other structural benefits afforded by research studies [4]. Although our participants did not describe feeling pressured to participate in research to gain access to medical care, their perception of enhanced access raises questions about whether healthcare access should be presented as an explicit “benefit” of study participation in the consenting process. Additionally, expectations of enhanced access may not always be met. Understanding what forms of support participants anticipate receiving from a study, and the extent to which those expectations will be met, could ensure that consent is truly informed.

Although the UARTO study protocol indicated that RAs should refer health-related questions to the clinic staff, health information was still conveyed through routine interactions. Participants often viewed RAs as health authorities who they could easily approach to receive a wide range of health advice. Additionally, despite the study’s intention to observe (not improve) adherence, participants felt motivated to adhere to ART in order to maintain relationships with research staff and thus sustain the emotional support these relationships created, as we have described elsewhere [17]. The extent to which social support affected adherence is an important outstanding empirical question. Our results indicate that observational studies in which study-derived social support can affect observational outcomes may need to measure and account for these effects in order to validly interpret results.

Material support from the study, in the form of transportation stipends, meals and incentives, were both valued unto themselves and perceived as a form of commitment to participants’ well-being. Transportation costs often impede access to HIV-related care in sub-Saharan Africa [27], and transportation reimbursements have been found to improve HIV-related care [28]. Others have argued that transportation reimbursements or incentives (like soap in this study) function as a form of payment for research participation and data [29, 30]. Our participants tended to view transportation refunds, incentives, and meals as “gifts” rather than as payment. As in prior studies, participants attached feelings of care and connectedness to the tangible incentives and reimbursements they received [29]. The research ethics community has struggled with balancing incentives against the prerogative to avoid undue inducement for study participation [31]. Meanwhile, others have argued that providing material benefit is one of researchers’ ethical responsibilities in resource-limited settings [32]. Our results underscore the emotional valence of material support for participants. Although the emotional aspects of receiving support are are difficult to quantify, they merit researchers’ consideration when designing, conducting, and concluding studies that are ethical and culturally sensitive.

We also found that the UARTO study created an environment in which participants could instrumentally support each other. The formation of a community savings group that included UARTO participants, as well as the relationships that study participants formed with each other, existed outside the realm of typical study benefit analysis. Nonetheless, these groups and social connections were an important source of material support for participations in UARTO. Investigators should remain vigilant for inter-participant support systems after studies commence, since these systems may have a tangible effect on participants’ willingness to enroll and remain in studies. Moreover, illuminating the social support networks that grow out of longitudinal studies is vital to understanding the context in which research data are generated, and the broader effects of research studies on the communities they take place in. Empirically, the longevity of these organically-formed groups warrants investigation, particularly after research studies end.

Participants interpreted both intended and unintended support as indications of care, and as cornerstones of their relationships with research staff. The importance of relationships that study participants form with research staff has been recognized previously, and participant-provider relationships have been incorporated into a model of factors affecting HIV adherence trial retention [33]. In qualitative research from sub-Saharan Africa, others have described how health-related and socioeconomic needs of research participants conflicted with research staff’s roles as objective data collectors [2–4]. Our findings provide evidence from the vantage point of study participants: rooted in durable relationships with research staff, UARTO participants experienced sustained emotional support from participation. This support helped to mitigate salient psychosocial challenges in their lives, such as HIV stigma.

Relationships with other study participants also provided substantial emotional support. HIV-related stigma and non-disclosure of serostatus have been identified as barriers to accessing social support in Uganda and elsewhere [10, 34, 35]. The UARTO study created a community of participants who had disclosed to each other while waiting “on the bench” for study visits. In this setting, participants could share stories, encouragement, and advice for living with the physical and psychological tolls of HIV.

The range and depth of social support that UARTO participants received have three important implications for longitudinal study design and conduct in sub-Saharan Africa with stigmatized populations, as well as for longitudinal study participants more broadly.

- Altered study behaviors and the Hawthorne effect. Although researchers acknowledge the potential to influence behavior through observation (namely, a Hawthorne effect [36]), our results indicate that social support received through the study may be a critical driver of (adherence) behavior modification. To understand the effects social support may have on behavioral study outcomes, researchers should be aware of the ways participants perceive protocolized incentives and stipends. The manner in which interactions with research staff generate unintended forms of support may promote behaviors that studies seek to observe.
- Managing study closure. The depth of social support that UARTO participants received through participation suggests that they may have much to lose when observational studies by necessity come to an end. Participants who lack resiliency may be particularly susceptible to stressors when they lose support resulting from studies [37]. Our results emphasize the need for researchers to understand and anticipate the psychosocial effects of study closure on participants. Researchers should also be prepared to manage participants’ expectations about durability of support. If negative consequence occur due to loss of social support at the end of a longitudinal study, investigators and ethics review boards should plan to inform prospective participants about this possibility.
- Risk/benefit. Social support derived from observational studies may function as a substantial benefit of participation. It may also create strong incentive to enroll and remain in research studies. These effects may be enhanced for individuals with stigmatized conditions living in resource-limited settings. Investigators and ethics review boards should consider how potential benefits of social support should be included into the overall risk/benefit calculus of studies. They could consider explicitly commenting on potential sources of support in consent forms and in discussions with potential participants.

We found that social support arose from standard, often obligatory study procedures (e.g., interaction between participants and RAs; provision of incentives). As such, our results do not suggest that investigators should intervene to manipulate social support that organically arises from observational studies. Rather, investigators should be prepared to address the consequences of its generation and potential removal, since it may form a core component of the experience of participating in an observational study, particulary among marginalized communities in resource-limited settings.

Our findings and these implications raise the need for additional empirical research. First, although a number of survey scales have been developed to measure social support, none address the unique features of study-related support that our participants described. Developing validated measurement tools could better equip investigators to quantitatively estimate and compare support arising from studies. Second, both qualitative and quantitative measures of social support could be used to estimate the effects of support on observational study outcomes. Measuring this potential source of bias could enable investigators to understand generalizeability of observational studies. Third, post-study research should address the longevity of study-related social support, and potential consequences arising from removal of support when studies end. Lasting effects of study-related support may be invisible while a study is ongoing. Likewise, changes in adherence behavior that arise as a consequence of study-derived social support may not last beyond study closure. Repeat measures of study-related behaviors after closure of intensive longitudinal observational studies could quantify the magnitude and kinetics of this effect. Fourth, additional research is needed to determine the range of social support across different populations, study types, and contexts. The magnitude of support received, and the ways in which support is perceived, undoubtedly vary between studies. For instance, study-derived social support may be less salient in research with non-marginalized populations, with populations without substantial resource constraints, or in short-term research that does not foster close relationships between staff and participants. The ability to plan for the effects of study-derived support hinges upon further exploration of this phenomenon in different settings and populations.

The limitations of our study should be noted. Interviews were conducted with a homogenous population of PLWH in southwest Uganda, and we did not purposefully sample participants to enable comparisons by gender, age, or other potentially relevant characteristics. We only interviewed individuals who were actively enrolled in UARTO, and therefore we were unable to assess baseline amounts of social support that non-participant PLWH receive from their communities and the ISS clinic. Despite efforts to distinguish our team from the UARTO study, the interviewer may have been perceived as being affiliated with the UARTO study, potentially skewing interview responses towards what participants thought would be socially desirable.

In conclusion, the range and depth of social support we identified have important implications for future observational study conduct. Our participants described the instrumental and emotional benefits that may arise from a study conducted with a vulnerable population in a resource-limited setting. Anticipating and examining these externalities of study participation will be vital for understanding the outcomes of observational studies, conceptualizing the choices that motivate study enrollment and retention, and mitigating any unintended consequences when studies must of necessity come to an end.

## Supporting information

S1 File Interview guides. (PDF)
