# Supplementary material for: “The research assistants kept coming to follow me up; I counted myself as a lucky person”: Social support arising from a longitudinal HIV cohort study in Uganda
Source: PLoS One. 2022 Jan 25;17(1):e0262989. doi: 10.1371/journal.pone.0262989 (PMC8789171; doi:10.1371/journal.pone.0262989)
Supplement: S1 File — (PDF) [file pone.0262989.s001.pdf]

EAM Ethics – Initial Exploratory Interview Guide

We are interested in learning more about your experience with Wisepill. There are no right or wrong answers. We just want to know your opinions and thoughts.

Please tell me about Wisepill. What does the Wisepill device do?

Probes: Who uses the information? How does that make you feel?

What do you like about Wisepill?

Probes: Ways it helps you?

What don't you like about Wisepill?

Probes: Ways it harms you?

What would you change about it?

Probes: Changes in the way it looks or works? Changes in how the data is used?

Tell me about being a participant in UARTO. What do you like about this?

Probes: Support from study staff? Ways it has affected your adherence?

What do you dislike about this?

Probes: Potential for others to know about your involvement/HIV status?

Is there anything you would like to tell me about the Wisepill device?

EAM Ethics—Initial Exploratory Interview Guide

Nitwenda kwongyera kwega ebikwatirine nokukozesa kwawe kw'akacuupa ka wisepill. Tihariho okugarukamu okuhikire ninga okugwire. Nintwenda kumanya entekateka n'edeeba yawe.

Bambe ngambira aha bikwatirine n'akacuupu ka wisepill. Akacuupa ka wisepill nikakoraki?

> Probe: Noha orikukozesa amakuru agarikwoherezibwa akacuupu ka wisepill? Eki nikituma wahurira ota?

Niki ebyorikukunda aha wisepill?

> Probe: Emiringo eyeri kukuyambamu?

Niki eby'otarikukunda aha wisepill?

> Probe: Emiringo eyeri kukukora kubi?

Niki ebi wakubasa kuhinduura aha wisepill?

> Probe: Okuhindura oku erikushisha nari erikukora. Okuhindura oku amakuru garikukozesibwa.

Ngambira ahabikwatirine nokwejumbira omukucondooza kwa UARTO. Niki ebyorikukunda omukwejumbira oku?

> Probe: Obuhwezi okuruga omu bacondoozi bomushomo. Emiringo ey'okumira emibazi yawe ehindukiremu.

Niki eby'otarikukunda omukwejumbira oku?

> Probe: Okutuma abantu bamanya ok'oyemerire omubikwatirine n'akakooko ka sirimu.

Hariho ekindi eky'orikwenda kungambira ekikwatirine n'akacuupa ka wisepill?

Qualitative Interview Guide - EAM Users

<Appropriate greeting for the season>

How are you?

We are interested in hearing your thoughts and opinions about the Wisepill device, and what it's like to use it. We want to find out what you like and don't like about it. Our goal is to help make sure that people who use the Wisepill device and other similar devices in the future have the best experience possible. There are no right or wrong answers to these questions.

Please tell me about the Wisepill device you are using for your pills. What does it do?

What do you like about using Wisepill?

What do you not like about using Wisepill?

> Probe: What would you change about using Wisepill (e.g., appearance, how it works, convenience, other features)?

[Dependence]

How has Wisepill helped you take your pills as directed, if ever? If no help, ask why not?

If UARTO takes the Wisepill device from you, how would it affect the way you take your medications? How would it affect your health?

What do you think the researchers should do with Wisepill when the study ends? Why?

[Privacy and Confidentiality]

Where do you keep the device? How do you travel with it?

The Wisepill device can sometimes draw attention from other people. Tell me about a time when someone noticed the Wisepill device or you using Wisepill.

> If Wisepill has been seen, probe: How did it make you feel? Did anything happen? Did it matter that you are using Wisepill for HIV medications, rather than other types of medications (for instance, malaria, high blood pressure or diabetes)? If so, why (i.e., to get at stigma)?

> If Wisepill has not been seen, probe: Are you trying to keep it secret? If so, how and why (i.e., to get at stigma)? How would you feel it someone saw it?

As you know, Wisepill records when you open it to take your pills and sends this information to researchers. Are you concerned about the opinions researchers form based on this information? If so, why or how?

How do you feel about sending information about your HIV medications over a cellular network?

> Probe: Are you concerned that people other than researchers or your doctor might learn about this information?

Do you have concerns about people learning other information from the Wisepill device besides when you take your pills? For instance, the time you take your pills could reveal or suggest other things about you like where you are or what your daily routine is.

[RA to provide reassurance as needed]

[**Trust in relationships]**

Imagine that you miss a dose of your medication—would you tell your doctor about it?

> Probe: What would happen? Reasons for and against disclosing this information, the perception of need to tell doctor, harms or benefits.

Imagine the study researchers knew about you missing a dose of your medication because of the Wisepill device.

> Probe: How would you feel? How would it affect your relationship with the study researchers or your research assistant?

In the future, Wisepill may be used for patients getting regular clinical care (that is, not in research studies) and this information could affect the relationship between patients and their doctors.

How do you think seeing information from your Wisepill device would affect your relationship with your doctors?

> Probe: Do you think it would change the level of trust in the relationship? Would it cause you to doubt the doctor or the doctor to doubt you?

[**Autonomy**]

As you know, when using Wisepill, researchers or doctors know when people don't open the device to take their medication. Has this fact changed your mind about deciding when to take or not take your HIV medication? If so, how?

How does Wisepill affect the way you feel about taking HIV medications?

> Probe: Does it cause feelings of obligation or resentment? Feelings of relief or support? Connection with the clinic?

What responsibilities do your doctors have to help you take your medications? What responsibility do UARTO researchers have?

Has using Wisepill ever caused you to change your plans (for example, travel plans)? Tell me about the most recent time this happened. How did that make you feel?

Participation in Research

Please tell me about your participation in the UARTO study.

Please tell me about your interactions with your RA.

Have visits related to using Wisepill (e.g. battery change visits or visits when the device did not record you taking your medication) resulted in any positive experiences? What about negative experiences? If so, please describe.

How has participation in UARTO changed the way you think about yourself and your health, if at all?

> Probe: caring (if brought up)

What has using Wisepill changed the way you think about yourself and your health, if at all?

[**Other questions]**

Who do you think should use Wisepill? Why?

> Probe: Should they be limited to certain people or conditions, like HIV? Should everyone taking medicines use Wisepill?

Should Wisepill be part of regular clinical care or should people have to ask for it?

Is there anything else you think I should know about using the Wisepill device?

Qualitative Interview Guide - EAM Users (Runyankole Version)

Ori ota? Eka yaawe eryeta?

Nitwetenga kuhurira ebitekateko hamwe nentekateka yaawe ahakacupa ka wisepill, hamwe nokukiri kukakozesa. Nitwenda kumanya ebyorikukunda hamwe nebyotakukunda ahakacupa aka. Ekigyendererwa kyeitu nokurebeka ngu abantu abakukozesa akacupa ka wisepill nari obundi bwoma nkobwo nyentsya bagyenderwa kurungi nkokukirikubasika. Tihariho bigarukamu ebigwire nari ebihikire ahabibuzo ebi .

Ngambira ahakacupa ka wisepill akokukozesa ahabwomubazi gwawe. Nikakoraki?

Nokundaki ahakukozesa wisepill?

Niki ekyotakukunda ahakukozesa Wisepill?

Probe: Niki ekiwakubaasa kuhindura ahakukozesa Wisepill?

[**Okukuma Ekihama]**

Akacupa ka wisepill obumwe nikareta okwecengyera kuruga ahabandi bantu. Ngambira obwomuntu areeba akacupa ka wisepill nari orikukakozesa.

Probe: Okahurira ota? Hine ekibi ekyabireho? Hine ekirungi ekyabireho? Kikagira enshonga yoona kugira ngu nokozesa wisepill ahabwemibazi yakakooko ka sirimu, kwihaho nari omumwanya gwendijo mibazi (ekyokureberaho, omushwija, puresha nari shukari)?

Wagambire aha kacupa ka wisepill hamwe neka yaawe nari abandi omuka yaawe?

Probe: Ahabwenki?

Nkokworikumanya, akacupa ka wisepill nikahandika obuwakingura kumira emibazi yaawe kandi kasindika obutumwa ahabacondoza. Nikibasika kusindika amakuru aga ahabashaho nari abandi bakozi bebyamagara, kwonka eki tikiriyo nikikorwa omumushomo gwa UARTO. Oyerarikirire okwabacondoza baratekateke nari okubarasharemu begamire amakuru aga?

Probe: Nohurira ota ahakyo kusindikisa za karimagyezi amakuru gaawe agemibazi yakakoko kasirimu, ekyokureberaho, kurabira aha netiwaka yesimu? Oyine okwerarikirira kugira ngu abandi bantu batari bacondoza babaasa kureeba amakuru aga?

Noha owokutekateka ashemereire kureeba wigura akacupa ka wise pill? Hariho omuntu otashemereire kugireba?

Probe: Ahabwenki?

Oyine enshonga ahabantu kumanya amakuru agandi kuruga aha kacupa wisepill kwihaho obworikumira obujuma bwawe, nkahori nari ebyokukora burijo?

[RA to provide reassurance as needed]

[**Obwesigwa]**

Ngambira ahabwire obwoyebwa doozi zawe zomubazi gwakakooko ka sirimu kandi abakozi bomushomo bakakimanya ahabwakacupa ka wisepill.

> Probe: Okahurira ota? Kikaretaho mpindukaki kakwatekawe nabacondoza nari nabakozi bomushomo?

Yensya, Wisepillebaasa kukozesibwa ahabwa abarweire abokutunga obujanjabi bwaburizooba (kitari ahabwemishomo yokucondoza) kandi amakuru aga gabaasa kureta empinduka aha kakwate akari ahagati yabarweire hamwe nabashaho baabo.

Tugambire ahakakwate kawe hamwe nabashaho baawe.

Probe:Noyesiga ebibarikugamba? Nogira ngu nibesiga ebigarukwamu byawe ahabwebibuzo byabo? Nogira ngu nibakuha amakuru gahikire?

Ngambira obuwabanganisa ekyomushaho yakugambire.

Ngambira obwosisirwa ngu omushaho yabanganisa ekiwagamba.

Nohurira ota ahakyabo kureba amakuru kuruga ahakacupa kawe ka wisepill?

> Probe: Obusaasi hamwe namagoba. Eki kibaasa kuhindura orwingano rwokubakukwesiga (munonga nari kukye)?

Enyima, kuwakuba otamira mubazi gwawe, okakigambiraho omushaho? Hakabaho ki?

> Probe: Enshonga zokwenda nobutenda kworeka amakuru aga. Entekateka yokwenda kugambira omushaho.

[**Obugabe]**

Obumwe abantu nibasharamu obutamira emibazi yaabo ahabwenshonga zitari zimwe na zimwe. Ngambira obwosharamu obutamira doozi yawe emwe nari nokurengamu yomubazi gwaawe gwa kakooko ka sirimu.

> Probe: Niki ekyakuteire omuhimbo kusharamu otyo? Okehurira ota aharyekyo? Hakabahoki obwo? Heine obuzibu bwabeireho?

Nkokworikumanya, waba nokozesa wisepill, abacondoza nari abashaho nibamanya obwabantu batigura kacupa kabo kumira emibazi yaabo. Eki heine okukihindwire okusharamu kwawe obwokumira nano obwotakumira mibazi yawe ? kyaba kiri ekyo, nibata?

Akacupa ka wisepill nikareta mpinduka ki ahakworikuhurira ahakyokumira emibazi?

Probe: Okuhurira obuvunanizibwa nari okweyanga? Okuhurira nohwerwa nari nobangirwa?

Nohurira obuvunanizibwa bwokumira emibazi yaawe? Abashaho baawe nari abacondoza beine buvunanizibwaki omukukuyamba kumira emibaziyawe? Wisepill eyine empinduka yekureta ahakworikuhurira? Kwerabe ego, bata?

[Okwegamira]

Ngambira omurundi obwokozesa wisepill ekakuyamba kumira emibazi yaawe nkokuyakuragirwe, kwerabe eriho.

Probe: Nibintuki/obukoryo ki oyihireho wisepill ebiwabire orakozise kukuyamba kumira?

Kuwakuba otakine wisepill, okworikumira emibazi yaawe kukahinduka? Kerabe ego, bata?

Notekateka ngu abacondoza bashemereire kukoraki na wisepill omushomo ogu gwaheza kuhwa?

Probe: Batware obucupa kuruga ahabantu abokubukozesa? Notekateka oshemereire kuguma nakoma aka?

[**Ebibuzo ebindi]**

Notekateka noha oshemereire kukozesa Wisepill? Ahabwenki?

Probe: Notekateka zishemereire kurekwa ahabantu abamwe nari embera ezimwe, nka akakooko ka sirimu?

Burikimwe kurinikibasika, buryome orikumira emibazi akahirwe akacupa ka wisepill? Ahabwenki?

Abantu beine kubuza ngubabahe wisepill, nari kashemeire kutekwa ahabujanjabi?

Noreeba ota wisepill erikukozesibwa aheeru yomushomo gwa UARTO?

Hariho ekindi ekyokutekateka nshemereire kumanya ahakukozesa akacupa ka wisepill?

Qualitative Interview Guide – EAM Non-Users

<Appropriate greeting for the season>

How are you?

When you joined the UARTO study, you were asked if you would use a device called Wisepill. This device records when people open it to take their medications. As you know, you decided not to use the device. We would like to hear more about why you did not want to use the device and what you think about the device. We respect your decision and just want to know more about it.

Please tell me why you decided not to use the Wisepill device?

What concerns, if any, did you have about the Wisepill device?

Tell me about what benefits you think might take place from using Wisepill. Tells me about harms that might occur.

[Dependence]

Do you have a system to help you take your medications on time? If so, what would happen if you could no longer use that system?

Have you or someone you know become dependent upon reminders (like a pill box), phones, or other strategies to take medications? What aspects of these reminders make people dependent upon them? Can you think of instances in which becoming dependent on the reminders has been harmful in some way?

How did your adherence change when you stopped using Wisepill?

[**Privacy and Confidentiality]**

The Wisepill device can sometimes draw attention from other people. How did this fact affect your decision not to use it?

Please describe a time, if any, when you were concerned about the privacy of your clinic medical information, especially relating to your HIV.

> Probe: Why were you concerned? What happened? Did anything reassure or not reassure you?

How do you feel about sharing your HIV medication information?

> Probe: With your family or friends. With your doctors. With UARTO researchers.

How would you feel if your doctors or researchers could tell if you didn't take your medicines?

Probe: Harms, benefits

Do you have any health-related information that you feel doctors, other health professionals or researchers should not know about?

[Trust in relationships]

Imagine that you miss a dose of your medication—would you tell your doctor about it?

Probe: What would happen? Reasons for and against disclosing this information, the perception of need to tell doctor, harms or benefits.

In case you miss taking your medication, how would you feel if UARTO researchers got to know about it before you told them? Do you think this would affect the way they treat you? What about if your doctors got to know about it before you told them?

In the past, if you have not taken your medication, did you tell your doctor about it? What happened?

Probe: Reasons for and against disclosing this information. Perception of need to tell doctor.

Do you think UARTO researchers would trust you more, less, or the same if they could tell when you had taken your medications and when you missed a dose? How do you think would this change your relationship with them? What about if your doctors could tell when you had taken your medications?

[**Autonomy]**

Sometimes people decide not to take their medications for a variety of reasons. Please tell me about a time when you decided not to take one or more doses of your HIV medication?

Probe: What motivated your decision? How did you feel about it? What happened then? What were the consequences, if any?

When using Wisepill, researchers or doctors know when individuals don't open the device to take their medication. Would this fact influence the way you take your HIV medication?

Probe: How/why? How would you feel?

Have you ever been reminded to take a dose of your medication? If so, what happened? How did you feel?

Probe: Resentment, support; who or what reminds the interviewee

What responsibilities do your doctors have to help you take your medications? What responsibility do UARTO researchers have?

Participation in Research

Please tell me about your participation in the UARTO study.

Please tell me about your interactions with your RA.

Did visits related to using Wisepill (e.g. battery change visits or visits when the device did not record you taking your medication) resulted in any positive experiences? What about negative experiences? If so, please describe.

How has participation in UARTO changed the way you think about yourself and your health, if at all?

Probe: caring (if brought up)

[**Other questions]**

Who do you think should use Wisepill?

Probe: Should they be limited to certain people or conditions, like HIV?

Should people have to ask for them? Should they be part of regular clinical care?

Probe: Differences for people taking HIV medications versus other types of medications.

Is there anything else you think I should know about using the Wisepill device?

Qualitative Interview Guide – EAM Non-Users (Runyankole Version)

Oryota? Eka yaawe eryeta?

Enyimaho, washabwa kutaha omumushomo ogu (gukwetwa UARTO) ogurikukozesa akacupa kakwetwa Wisepill. Akacupa aka nikahandika abantu kubakwigura kumira emibazi yaabo. Nkokworikumanya, oshaziremu obutejumba omumushomo ogu. Nitwenda kwongyera kumanya ahabwenki wabire otarikwenda kwejumba kandi nebyokutekateka ahakacupa. Nituteka ekitinisa omukusharamu kwawe kandi nitwenda kwongyera kumanya ahakusharamu okwo..

Ngambira ahabwenki washaziremu obutejumba omumushomo gwa UARTO?

Ninshongaki, kuzirabe zabire ziriho eziwabeire oyine aha kacupa ka wise pill?

Okagambaho neka yaawe nari nabandi omuka yaawe ahakyokushabwa kuza omumushomo gwa UARTO nari kukozesa wise pill?

Ngambira aha magoba agokutekateka kuba gari omukukozesa wisepill. Ngambira ahabuzibu oburikubaasa kubaho.

[**Okukuma ebihama]**

Akacupa ka wisepill kabaasa kureta okwecengyera kuruga ahabandi bantu. Enshonga egi ekareta eta empinduka omukusharamu kwawe obutejumba omumushomo gwa UARTO?

SHoborera obwire, kuburabe buriho, obwoyerarikirira ahabihama bya makuru gaawe gokuraguza aha kirinika, namunonga ebikwatirine nakakooko ka sirimu.

> Probe: Ahabwenki wayerarikirire? Hakabahoki? Hine ekyakuhamize nari ekitarakuhamize?

Nohurira ota ahakyokubagana amakuru gaawe gobujanjabi bwakakooko ka sirimu?

> Probe: Neka yaawe nari na banywani bawe. Nabashaho baawe.

Okahurira ota abashaho baawe nari abacondoza kubakukugambira waba otamizire mubazi gwaawe?

> Probe: Akabi, amagooba

Oyine amakuru agokukwata ahamagara gaawe agokutekateka ngu abashaho nari abandi bakozi bebyamagara nari abacondoza batashemereire kumanya?

[Obwesigwa]

Ngambira ahakakwate kawe nabashaho baawe.

Probe: Noyesiga ebibarikugamba? Nogira ngu nibesiga ebigarukwamu byawe ahabwebibuzo byabo? Nogira ngu nibakuha amakuru gahikire?

Ngambira obuwabanganisa ekyomushaho yakugambire.

Ngambira obwosisirwa ngu omushaho yabanganisa ekiwagamba.

Kukyakubaho okabura kumira omubazi gwaawe, okahurira ota omushaho waawe kuyakukimanya otakamugambire? Notekateka ngu ekikibaasa kuhindura okwomushaho akukutwariza?

Enyima, kuwakuba otamira mubazi gwawe, okakigambiraho omushaho? Hakabaho ki?

> Probe: Enshonga zokwenda nobutenda kworeka amakuru aga. Entekateka yokwenda kugambira omushaho.

Notekateka ngu abashaho baawe bashemereire kwongyera, kucendeza nari bagumizaho kukwesiga, baba nibabaasa kumanya yaba wamizire nari otaramizire emibazi yaawe? Nogira eki kikahindura kita akakwate kawe nabo?

[**Obugabe]**

Obumwe abantu nibasharamu obutamira emibazi yaabo ahabwenshonga zitari zimwe na zimwe. Ngambira obwosharamu obutamira doozi yawe emwe nari nokurengamu yomubazi gwaawe gwa kakooko ka sirimu.

> Probe: Niki ekyakuteire omuhimbo kusharamu otyo? Okehurira ota aharyekyo? Hakabahoki obwo? Heine obuzibu bwabeireho?

Waba nokozesa wisepill, abacondoza nari abashaho nibamanya obwabantu batigura kacupa kabo kumira emibazi yaabo. Eki heine okukyakuhindwire engyeru yokumiramu omubazi gwawe gwakakooko ka sirimu?

> Probe: Bata/ahabwenki? Okahurira ota?

Barakwijukizeho kumira emibazi yaawe? Kwerabe ego, hakabahoki? Okahurira ota?

> Probe: Okuhurira obuvunanizibwa nari ohwerwa; noha nari niki ekikwijutsya oworikubuza

Nohurira obuvunanizibwa bwokumira emibazi yaawe? Abashaho baawe beine buvunanizibwaki omukukuyamba kumira emibaziyawe?

[Okwegamira]

Oyine enkora ekukuyamba kumira emibazi yawe omubwire? Kwerabe ego, hakabahoki kuwakuba otakikozesa enkora egyo?

Heine nari omuntu oworikumanya kwegamira ebyijutsyo (nkobubokisi bwobujuma), esimu, nari ezindi nkora zokumira emibazi? Nibintuki ahabyijusyo ebi ebikuretera abantu byegamira? Nobaasa kutekateka ahabwire obwokwegamira ebyijutsyo kibeire ekyobuzibu omugyeru emwe?

[**Ebibuzo ebindi]**

Notekateka noha oshemereire kukozesa Wisepill?

> Probe: Notekateka zishemereire kurekwa ahabantu abamwe nari embera ezimwe, nka akakooko ka sirimu?

Abantu beine kubuza ngubabahe wisepill, nari kashemeire kutekwa ahabujanjabi?

Burikimwe kurinikibasika, buryome orikumira emibazi akahirwe akacupa ka wisepill? Ahabwenki?

> Probe: Entaniso yabantu abokumira emibazi yakakooko ka sirimu hamwe nebindi bika byemibazi.

Hariho ekindi ekyokutekateka nshemereire kumanya ahakukozesa akacupa ka wisepill?

Qualitative Interview Guide – UARTO Staff

How are you?

As you know, Wisepill and other devices like it are being used to measure adherence. We're conducting a study to investigate the ethics of using these devices. Since you work so closely with the people who use Wisepill, as well as the devices themselves, we'd like to hear your thoughts about them. There are no right or wrong answers, and your responses will be kept confidential. They will not affect your work in anyway.

What is your overall impression of Wisepill?

Probe: Likes? Dislikes?

Tell me about a time when there was a technical challenge with Wisepill? How did the Wisepill user react? How did you feel about it?

What benefits have you seen for participants using Wisepill?

What harms have you seen for participants using Wisepill?

Please describe any concerns Wisepill users have mentioned to you about the device or participation in the adherence monitoring aspects of UARTO.

[Privacy/Confidentiality]

We're interested in knowing what Wisepill users think about sharing information with their providers (which we call "privacy") and with people not involved in their care, like neighbors (which we call "confidentiality"). In particular, we want to know how Wisepill has affected the way other people think about the participants.

What kinds of discussions about privacy and confidentiality have you had with UARTO participants?

Probe: When have you had these types of discussions? What questions have they asked?

Have you seen any instances in which people felt that their personal information was not handled privately or confidentially because of Wisepill? What happened? How did they react?

Have you noticed instances in which *you* didn't feel that participant's information was handled privately or confidentially? What happened? What did you do?

How does HIV affect the need to keep health information private and confidential? For instance, would it be different for other health conditions like hypertension or diabetes?

Do you think UARTO participants' attitudes about privacy and confidentiality changed during the study? If so, how?

[Data Security]

Because Wisepill collects and transmits data electronically, people outside of UARTO could potentially have access it.

What do you tell UARTO participants about how their Wisepill data is transmitted, stored, and/or handled? What concerns about their Wisepill data have they mentioned to you?

Have you encountered any scenarios in which you thought Wisepill data might not have been secure? What happened?

[**Ancillary Care Obligations]**

As a researcher, do you have a role in supporting participants to take their medications? If so, what?

> Probe: How does this change based on the study design (for example, observational versus interventional studies)?

Have participants ever expressed the expectation that you will help them in taking their medications? If so, please tell me what they said and what you did.

Have you noticed that participants' adherence changes while they use the Wisepill? If so, in what ways?

What do you think should be done with the Wisepill devices at the end of UARTO? Do you think the study has any obligation to support participants? If so, what would be the biggest challenges to meeting these obligations?

[Other questions]

What do you think would be the biggest challenges to expanding Wisepill for use with all patients at the ISS clinic?

If Wisepill adherence monitoring became available for all HIV patients, should patients be able to choose whether or not to use the device? If so, how should the option to participate be presented?

Is there anything else you think I should know about the Wisepill device?
